# Annexins induce curvature on free-edge membranes displaying distinct morphologies

**DOI:** 10.1038/s41598-018-28481-z

**Published:** 2018-07-09

**Authors:** Theresa Louise Boye, Jonas Camillus Jeppesen, Kenji Maeda, Weria Pezeshkian, Vita Solovyeva, Jesper Nylandsted, Adam Cohen Simonsen

**Affiliations:** 1 University of Southern Denmark (SDU), Campusvej 55, DK-5230 Odense M, Denmark; 2 Department of Physics, Chemistry and Pharmacy, University of Southern Denmark, Odense, Denmark; 3 Department of Biochemistry and Molecular Biology, University of Southern Denmark, Odense, Denmark; 4 Membrane Integrity Group, Unit for Cell Death and Metabolism, Danish Cancer Society Research Center, Strandboulevarden 49, DK-2100 Copenhagen, Denmark; 5 Department of Cellular and Molecular Medicine, Faculty of Health Sciences, University of Copenhagen, DK-2200 Copenhagen N, Denmark

## Abstract

Annexins are a family of proteins characterized by their ability to bind anionic membranes in response to Ca^2+^-activation. They are involved in a multitude of cellular functions including vesiculation and membrane repair. Here, we investigate the effect of nine annexins (ANXA1-ANXA7, ANXA11, ANXA13) on negatively charged double supported membrane patches with free edges. We find that annexin members can be classified according to the membrane morphology they induce and matching a dendrogam of the annexin family based on full amino acid sequences. ANXA1 and ANXA2 induce membrane folding and blebbing initiated from membrane structural defects inside patches while ANXA6 induces membrane folding originating both from defects and from the membrane edges. ANXA4 and ANXA5 induce cooperative roll-up of the membrane starting from free edges, producing large rolls. In contrast, ANXA3 and ANXA13 roll the membrane in a fragmented manner producing multiple thin rolls. In addition to rolling, ANXA7 and ANXA11 are characterized by their ability to form fluid lenses localized between the membrane leaflets. A shared feature necessary for generating these morphologies is the ability to induce membrane curvature on free edged anionic membranes. Consequently, induction of membrane curvature may be a significant property of the annexin protein family that is important for their function.

## Introduction

The annexin protein family is a group of Ca^2+^-dependent biomembrane interacting proteins that reversibly bind anionic phospholipid head groups. In cells, annexins function as membrane-membrane or membrane-cytoskeleton linkers involved in exocytosis, endocytosis, intracellular signalling, vesicle transport and membrane repair, but annexins also have extracellular activities in inflammation, coagulation and fibrinolysis^1^. In homo sapiens, 12 annexin family members (ANXA1-11, ANXA13) are found. The annexin core domain is conserved among all members of the annexin family and localized in the C-terminal region of the protein. It consists of four very similar repeats (eight in ANXA6), referred to as annexin repeats^2^. Each of the four annexin repeats contains 5 highly *α*-helical domains that forms a funnel-shaped pore in which Ca^2+^ binds^3,4^. The annexin core domain has the shape of a slightly curved disc with the membrane attachment sites located at the convex face^5^. In contrast to the conserved core domain, the N-terminal region is variable in length (few residues to >200 residues) and displays great amino acid diversity^2^.

Disruption of the plasma membrane poses a lethal threat to cells, which they cope with by activating their plasma membrane repair system to reseal the membrane. The repair system includes mechanisms to remove damaged membrane by excision or shedding, internalization by endocytosis and membrane fusion events^6^. Annexin family members are involved in plasma membrane repair where they appear to play specific functions by regulating membrane curvature and facilitating membrane fusion events. To this end, our recent results show that upon membrane injury, ANXA4 and ANXA6 translocate to the vicinity of membrane wound edges and here induce curvature and a constriction force, respectively, which helps pull the wound edges together for eventual fusion^7^. Moreover, both ANXA1, ANXA2 and ANXA5^8^ have been reported to be involved in cell membrane repair although their precise function in the repair response remains to be elucidated. Repair also involves recruitment of proteins that are non-homologous to annexin such as the ESCRT proteins^9^. ESCRT facilitates repair by vesicle shedding and it has been demonstrated that ESCRT III assembly is catalyzed by pre-existing negative membrane curvature^10^.

Various potential mechanisms have been proposed for annexin-membrane binding and annexin-mediated membrane aggregation. First, simultaneous monomeric annexin binding of the annexin core and the N-terminal domain was reported for ANXA1 and ANXA2. In the case of ANXA1, Ca^2+^ ions induce a conformational change leading to the presentation of the N-terminal domain that acts as a secondary membrane binding site essential for membrane aggregation^11–13^. Similarly, the N-terminal domain of ANXA2 was also reported to be important for membrane aggregation^14^, indicating that the N-terminal domain of ANXA2 may also act as a secondary membrane binding site. Another way for ANXA1 and ANXA2 to aggregate membranes involves forming hetero-dimers consisting of two annexins connected via the N-terminus by two S100 proteins^15–18^. Interestingly, a recent molecular dynamics study reports ANXA2 to induce negative curvature in Ca^2+^ dependent manner on a negatively charged membrane containing phosphatidylserine (POPS)^19^. Indeed, previous experiments have demonstrated inner vesicles from parental giant vesicles induced by the generation of membrane curvature from ANXA2^20^.

ANXA4 and ANXA5 differ from other annexins by self-associating into crosslinked trimers, hexamers and larger aggregates in a Ca^2+^ dependent manner on membranes or vesicles with negatively charged phospholipids^21–24^. We have demonstrated that ANXA4 via its trimerization induces curvature on phosphatidylserine containing membranes, eventually rolling up whole membrane patches^7^. In support of this mechanism, membranes aggregated by ANXA4 have shown a separation distance between the two bilayers compatible with two layers of membrane-bound ANXA4^24^. Similarly, self-associated ANXA5 maintains its bent shape upon membrane binding, suggesting that ANXA5 could also induce membrane curvature^25,26^. Although ANXA6 also forms aggregates^27^, it is unique in being the only annexin containing two annexin core domains, probably originated from duplication and fusion of the genes for ANXA5 and ANXA10^28^. Between the two core domains, ANXA6 has a flexible linker which implies that the protein can orient itself to cross-bind different membranes simultaneously^29^. We have previously identified ANXA6 to trigger in-plane constriction of membrane edges as well as shrinkage of supported membrane islands, possibly facilitated by this unique flexible linker^7^.

ANXA7 and ANXA11 are characterized by having the longest N-terminal regions of human annexins^30^. Like other annexins, ANXA7 self-associates on membrane surfaces and induces membrane fusion and aggregation^31^. Although the specific function of the N-terminal region of ANXA7 is yet to be established, deleting 100 amino acids negatively affects membrane binding, aggregation and fusion, indicating that the N-terminus is necessary for phospholipid binding^32^. Additionally, the N-terminal region of ANXA7 harbours the binding sites to other proteins including sorcin, galectin-3 and Apoptosis-linked gene-2 (ALG-2)^33–35^. Not much is known about ANXA11. Studies using recombinant mouse ANXA11 argues that ANXA11 like other annexins induces vesicle aggregation, possibly via its long N-terminal region^36^.

ANXA3 and ANXA13 are some of the least studied annexins. ANXA13 is considered as the founder genes of vertebrate annexins^37,38^. Similar to other annexins, ANXA3 binding to Ca^2+^ and membranes modulates protein conformation, suggesting that ANXA3 like other family members is activated by Ca^2+ ^^39^.

**Figure 1:**
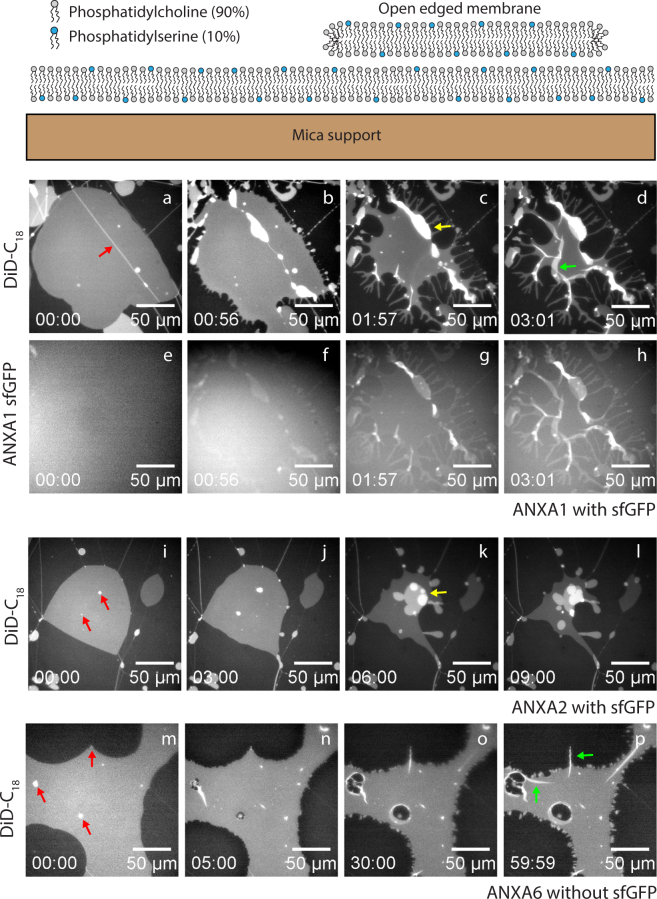
Typical configuration of a membrane stack prepared by hydration of a spincoated lipid film as used in the present study (top). The response of an open edged membrane patch is monitored by time-lapse fluorescence imaging after exposure to annexins. Sequence (**a–d**) and sequence (**i–l**) show the response of an open edged membrane (DiDC18) to exposure to ANXA1 and ANXA2 respectively. Contraction and blebbing from the patch is evident in both cases and folding is also evident for ANXA1. Sequence (**e–h**) shows the GFP channel (sfGFP-ANXA1) demonstrating binding of ANXA1 to the membrane. Red arrows indicate initial defect sites, the green arrow is a fold structure and the yellow arrows are blebs. Response of a membrane patch to ANXA6 (**m–p**). The membrane responds by modifying the free membrane edges and by creating folds as characterized by bright elongated structures. Nucleation of membrane holes inside the membrane patch is observed in parallel with folding. Concentrations: ANXA1-sfGFP: 46 nM, ANXA2-sfGFP: 58 nM, ANXA6: 50 nM.

The impact of annexin family members on membrane morphology and shape was previously evaluated using negatively charged vesicles^7,40^. Although some membrane binding and cross-linking between membranes can be observed in these systems, the generation of membrane curvature in vesicles can in some cases be restricted by the encapsulated volume and the associated membrane tension. As an alternative method, membrane shape changes without an area expansion can be observed in a solid supported membrane model composed of single and multiple membrane patches laying on a primary membrane, as shown in Fig. 1. This system can be formed upon hydration of a multilayered spincoated lipid film and is generally useful as an alternative to giant vesicles^41,42^. The membrane patches have the unique advantage of containing stable, free edges that have conformational freedom to bend away from the surface. The membrane is not strongly constrained by the support as the patch can also slide on the primary membrane. Here we tested a series of recombinant human annexins with respect to their effect on single membrane patches with free edges. The results can be grouped according to similarities in the morphology induced by the annexins. This grouping is found to agree well with a dendrogram of the proteins based on alignment of their full amino acid sequences.

## Results

### ANXA1, ANXA2 and ANXA6: Membrane blebbing and folding

Addition of recombinant ANXA1-sfGFP or ANXA2-sfGFP proteins both induce a similar type of response in anionic membrane patches with composition POPC,POPS (90%, 10%). The typical response of a membrane patch upon exposure to ANXA1 shows that two main morphologies are observed: Blebbing and folding (Fig. 1a–d). Blebbing is the initial response after short time scales (<1 min) and results in the generation of round vesicular structures emerging from the patch surface. The blebs are connected to the patch and are initiated at the patch surface at sites with structural defects such as the tube indicated by a red arrow (Fig. 1a). The blebs grow by drawing material from the patch which responds by retracting from the edge due to conservation of the area of the membrane patch. The folding morphology appears later (∼minutes) than the blebbing and is characterized by bright, linear protrusions with sharp edges (Fig. 1d). The shape of the folds is distinctly different from the round shape of vesicles and blebs.

Imaging of the patch in the sfGFP channel confirms that blebbing and folding is associated with binding of ANXA1 to the patch surface(Fig. 1e–h). In fact, the protein becomes incorporated into the bleb and fold structures as revealed by the strong fluorescence from these regions (Fig. 1h).

Membrane patches respond to ANXA2 in a manner that is qualitatively similar to ANXA1, showing the formation of blebs originating from the central region of the original patch surface (Fig. 1i–l). The small defects originally present, act as nucleation sites for a blebbing process upon addition of ANXA2 (Fig. 1i (red arrows)). Membrane area of the patch is pulled into the vesicular structures created at the defect sites while the outer boundary of the patch is retracted.

For both ANXA1 and ANXA2, most of the original patch area eventually becomes converted into blebs and fold structures over a timescale of less than 10 minutes. Negative controls where performed in the absence of Ca^2+^ and showed no response of the membrane patch to exposure to ANXA1 or ANXA2 confirming that annexin binding is calcium dependent (see Supplementary Fig. S5). Interestingly, the folding morphology is also induced by the family member ANXA6 (Fig. 1m–p). But for this particular annexin, only folding was observed and not blebbing or rolling. This fact indicates that the unique dimeric structure of ANXA6 containing two connected annexin core domains, restricts the membrane perturbation to the formation of folds. The time scale for formation of folds induced by ANXA6 is on the order of minutes and similar to the time scale for folding induced by ANXA1 and ANXA2. For ANXA6, folding is localized at the free edges of the membrane or at visible defects sites in the original membrane and can lead to membrane holes surrounded by folded edges (Fig. 1p).

### ANXA4 and ANXA5: Cooperative membrane rolling

**Figure 2:**
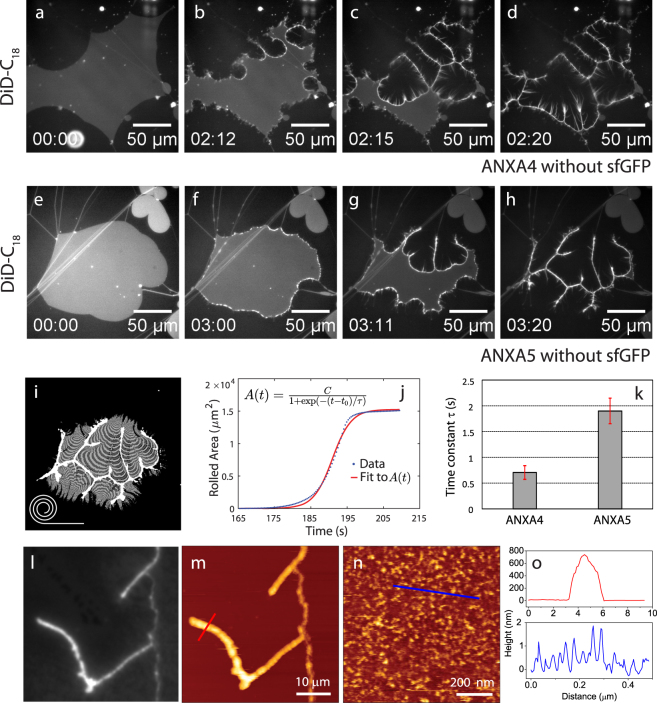
Response of membrane patches to exposure to ANXA4 (**a–d**) and ANXA5 (**e–h**) respectively. In both cases a complete roll-up of the membrane patch is observed as initiated from points on the free edges. Analysis of the rolling dynamics was performed by quantification of the incremental area reduction between image frames as shown graphically in (**i**). Alternating bright and dark grey bands (**i**) show the area reduction during rolling. The rolling dynamics plotted as the cumulative rolled area (**j**) and the time constant *τ* derived from fit to a logistic function (**k**). Histograms in (**k**) are based on n = 5 repeats. A logistic function was fitted to the rolled area (**j**) which provides a characteristic time scale *τ* for the rolling dynamics. Fluorescence image of a roll pattern induced by ANXA4 and the corresponding AFM topography image of the same region (**l,m**). AFM image of the primary membrane after exposure to ANXA4 showing patterns of bound ANXA4 (**n**). Line profiles of the AFM images (**o**). Concentrations: ANXA4: 43 nM, ANXA5: 44 nM.

The exposure of membrane patches to recombinant ANXA4 or ANXA5 induces strong curvature and membrane rolling as initiated from the free edges. The final stripe morphology of the patch in response to annexin exposure is highly similar for both ANXA4 and ANXA5. The mechanism of this process is roll-up of the bilayer, as previously documented for ANXA4^7^. A typical response shows that rolling starts at a few discrete points on the membrane edge and propagates inwards into the patch (Fig. 2a–h). The rolling process takes a few seconds, but is typically delayed by 1–3 min needed for annexin to reach the membrane surface by diffusion through the aqueous phase. The integrated fluorescence intensity of the bright bands after completion of rolling corresponds to the total intensity of the original patch, indicating that membrane material is not lost. During annexin mediated rolling, annexin monomers bind to one side of the membrane patch and trimerize into aggregates inducing a spontaneous curvature mediated by the curved disc shape of the protein binding region^23^. The consequence is that the elastic energy of the membrane/protein sheet becomes lower in the rolled state than in the original planar state. Note that rolling induced by ANXA4 and ANXA5 is mainly characterized by one major roll oriented parallel to the membrane edge. We refer to this as cooperative rolling. Negative controls for rolling were performed using the mutant ANXA4-Ca3mut having only one Ca^2+^ binding site and with Lact-C2-GFP, the PS binding domain of lactadherin. Both proteins displayed binding without rolling (for further details see Supplementary Fig. S5).

The rolling dynamics was quantified by image analysis (Fig. 2i–k). The total rolled area of the membrane patch follows a sigmoidal trend with respect to time (Fig. 2j). When fitted to a so-called logistic function, this yields a characteristic time scale *τ* for the rolling dynamics which is *τ*(ANXA4) = 0.71 ± 0.13 s and *τ*(ANXA5) = 1.90 ± 0.25 s.

The topography of membrane rolls after exposure to ANXA4 was measured with Atomic Force Microscopy (AFM) (Fig. 2m). The AFM corresponds well to the DiD lipid fluorescence image of the same region. AFM shows a diameter of the membrane rolls in the range of typically 500–800 nm while AFM scanning of the primary membrane reveals a disordered overlayer of ANXA4 proteins (Fig. 2n). This demonstrates uniform binding of ANXA4 to the membrane surface although strong adhesion of the primary membrane to the mica support prevents the primary membrane from rolling.

### Model of membrane rolling

**Figure 3:**
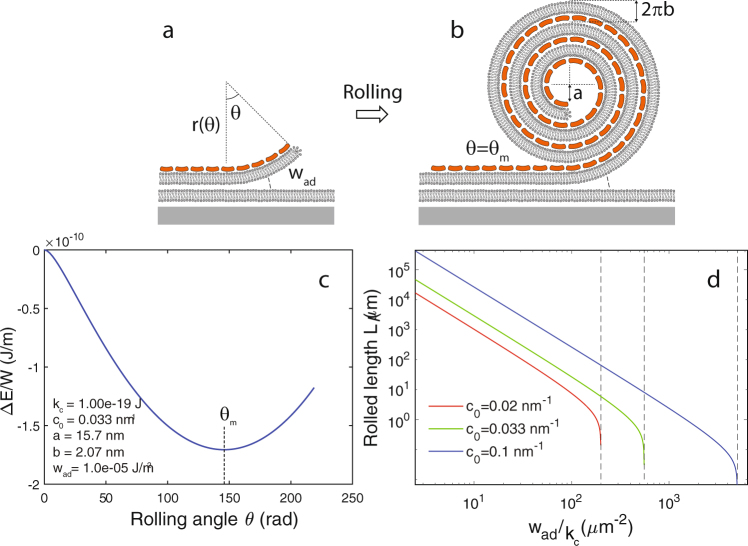
Theoretical model for rolling of a membrane patch induced by annexin binding. After initiation of rolling (**a**) the rolling proceeds until the roll radius becomes limiting (**b**). Plot of the energy change (equation 2) for roll formation as a function of the rolling angle *θ* (**c**). Rolling stops at the minimum *θ* = *θ*_*m*_. Plot of the rolled length as a function of the ratio *w*_a*d*_/*k*_*c*_ for three values of the spontaneous curvature *c*_0_ induced by annexin binding (**d**). Vertical dashed lines in (**d**) are the thresholds for rolling determined from equation (1).

A theoretical model was constructed to evaluate the energy change during rolling and to establish criteria for the initiation and stopping of membrane rolling (Fig. 3a,b). A planar bilayer patch resting on a primary supported membrane is considered as the initial state. Its adhesion energy to the underlying membrane is *w*_a*d*_ (J/m^2^) and the binding of annexin to the membrane induces a spontaneous curvature *c*_0_ (nm^−1^). The mechanical bending stiffness of the membrane is characterized by its *mean curvature elastic modulus k*_*c*_ (J). The model examines the formation of a roll of width *W* with elastic energy described according to the Helfrich hamiltonian^43^ and assuming that the membrane area is unchanged during rolling.

Rolling is initiated when the spontaneous curvature overcomes the adhesion to the support. This condition for rolling can be expressed as the inequality:

1 $$\frac{{k}_{c}}{2}{c}_{0}^{2} > {w}_{{\rm{ad}}}$$

Rolling will stop when the radius of the roll becomes so large that the curvature elastic energy can no longer overcome the adhesion to the support. The formation of a roll is modeled as an archimedean spiral: *r*(*θ*) = *a* + *bθ* (Fig. 3b). Here *r*(*θ*) is the radius of the spiral at the rolling angle *θ*, *a* is the radius of the inner roll and 2*πb* is the repeat distance between roll layers. The change in elastic energy Δ*E* becomes:

2 $${\rm{\Delta }}E=\frac{W{k}_{c}}{b}[\frac{1}{2}\,\mathrm{ln}(1+\frac{b}{a}\theta )+(\frac{{w}_{{\rm{a}}d}}{{k}_{c}}ab-{c}_{0}b)\theta +\frac{1}{2}\frac{{w}_{{\rm{a}}d}}{{k}_{c}}{b}^{2}{\theta }^{2}]$$

Minimization of Δ*E* in equation (2) with respect to *θ* and *a* yields the following expressions for the parameters in the final state of the roll:

Maximum roll radius *r*_*m*_:

3 $${r}_{m}=\frac{{c}_{0}+\sqrt{{c}_{0}^{2}-2\frac{{w}_{{\rm{ad}}}}{{k}_{c}}}}{2\frac{{w}_{{\rm{ad}}}}{{k}_{c}}}$$

The inner radius *a*:

4 $$a={[2\frac{{w}_{{\rm{ad}}}}{{k}_{c}}{r}_{m}]}^{-1}$$

The maximal rolling angle *θ*_*m*_:

5 $${\theta }_{m}=\frac{{r}_{m}-a}{b}$$

Rolled distance *L*:

6 $$L=a{\theta }_{m}+\frac{b{\theta }_{m}^{2}}{2}$$

The slope *b* of the archimedean spiral:

7 $$b=\frac{{r}_{m}^{2}-{a}^{2}}{2L}$$

The typical variation of Δ*E* with rolling angle *θ* using published values of *k*_*c*_ = 1 ⋅ 10^−19^ J^44^ and *w*_a*d*_ = 1 ⋅ 10^−5^ J/m^2 ^^45^ shows a minimum in Δ*E* occuring at an angle *θ*_*m*_ which is the maximum angle for which rolling is energetically favored (Fig. 3c). The corresponding maximum rolled length *L* plotted against the ratio *w*_a*d*_/*k*_*c*_ is shown for three values of the spontaneous curvature *c*_0_ (Fig. 3d). For comparison, values of *c*_0_ for Shiga toxin (*c*_0_ = 0.033 nm^−1^) and Cholera toxin (*c*_0_ = 0.028 nm^−1^) where recently reported^46,47^. If the ratio *w*_a*d*_/*k*_*c*_ is in the realistic range of 10–100 *μm*^−2^, then the model predicts rolling lengths *L* exceeding 1000 *μm* which is much larger than the characteristic size of membrane patches measured in this study. Thus, full roll-up of membrane patches is predicted as energetically feasible based on estimates in the present model. From equation (7) the layer spacing *d* = 2*πb* can be estimated using the experimentally measured values of *L* and *r*_*m*_. With a typical measured roll radius of *r*_*m*_ = 350 nm, rolled distance L = 30 *μm* and internal radius a = 15 nm, the inter-bilayer spacing becomes d $\simeq$ 13 nm. This can be considered a realistic value in the presence of an annexin spacer between the membranes inside a roll. It also compares well with our previously estimated spacing of 14 nm obtained by modeling the roll as concentric circles^7^.

### ANXA13 and ANXA3: Fragmented rolling

**Figure 4:**
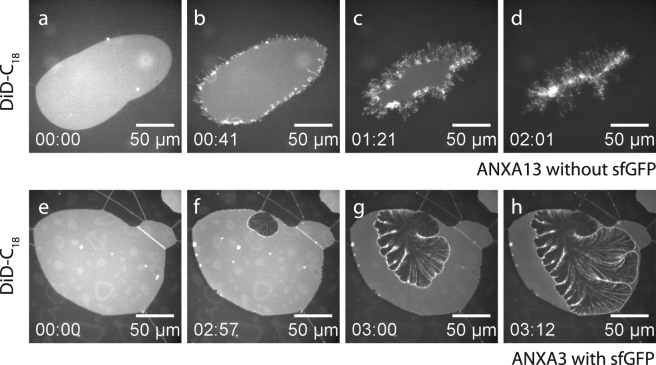
Response of a membrane patch to ANXA13 (**a–d**) and ANXA3 (**e–h**). For ANXA13, the membrane responds by formation of multiple thin roll structures initiated at the free edge that finally degrade the planar patch. For ANXA3, rolling also results in multiple thread-like rolls. Concentrations: ANXA3-sfGFP: 49 nM, ANXA13: 53 nM.

In contrast to the cooperative rolling induced by ANXA4 and ANXA5, a second type of rolling is observed in membranes exposed to ANXA3 and ANXA13, which we broadly denote fragmented rolling. While cooperative rolling is characterized by large single rolls oriented parallel to the membrane edge, fragmented rolling is characterized by multiple thin rolls oriented mainly perpendicular to the membrane edge and being often branched and irregular. An example of fragmented rolling by ANXA13 shows the membrane edge gradually being transformed into multiple thin rolls partly suspended away from the surface and fluctuating into solution (Fig. 4a–d). Fragmented rolling induced by ANXA13 occurs over a timescale of 60–90 sec.

Fragmented rolling induced by ANXA3 is slightly different and is characterized by multiple thin rolls forming a branching pattern (Fig. 4e–h). Fragmented rolling induced by ANXA3 occurs over a timescale of 10–20 sec and is thus faster than ANXA13. GFP imaging of ANXA3-sfGFP (see Supplementary Fig. S9) shows that annexin becomes incorporated into the branching pattern, thus supporting that the fragmented structures are compatible with a rolling mechanism.

### ANXA7 and ANXA11: Rolling and lens formation

**Figure 5:**
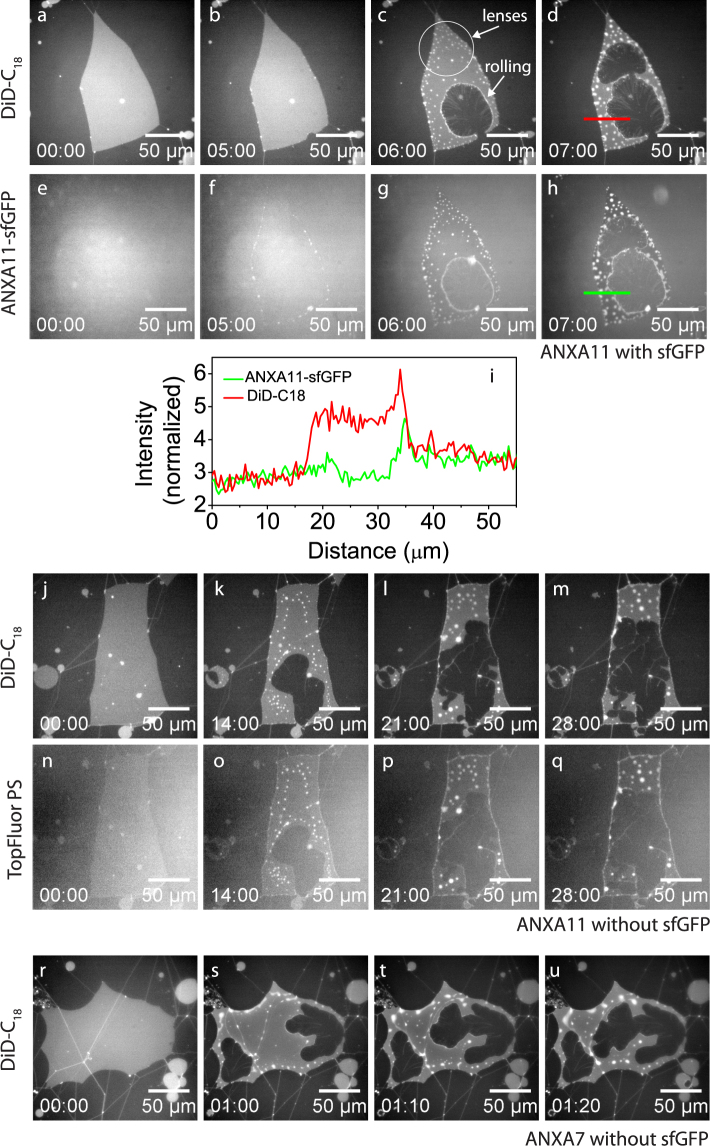
Response of membrane patches to ANXA7 and ANXA11 which both induce very similar types of membrane modifications. Imaging of the DiD membrane probe shows reduction of the patch area and formation of characteristic bright lens structures for ANXA11-sfGFP (**a–d**) and for ANXA11 (**j–m**). Imaging in the GFP channel of ANXA11-sfGFP shows that ANXA11 is being incorporated into the lens structures (**e–h**) and depleted from the membrane surface (**i**). TopFluorPS labeling shows that the anionic PS lipid localizes to the lens structures (**n–q**). Comparison with results for ANXA7 (**r–u**) displaying both reduction in area and lens formation, equivalent to the observations for ANXA11. Concentrations: ANXA11-sfGFP: 31 nM, ANXA11: 32 nM, ANXA7: 38 nM.

ANXA7 and ANXA11 are characterized by inducing a bimodal and highly similar response in anionic membrane patches. The first of the two modes is the rolling morphology as shown for ANXA11 (Fig. 5c). The observed rolling is mainly of the cooperative kind with small traces of fragmented rolls perpendicular to the membrane front. The second type of morphology induced by ANXA7 and ANXA11 is the formation of dynamic bright aggregates in the membrane which we denote *lenses*. The lens structures are only observed for ANXA7 and ANXA11 and appear as bright dots on the membrane patch (Fig. 5c). Lenses are predominantly nucleated near the edges of the membrane patch and are initially small and probably below the resolution limit when nucleated. Visible coarsening and fusion of the lenses take place over time, as visible in the time-lapse sequences for ANXA7 and ANXA11 (Fig. 5 and Supplementary Fig. S8). Imaging of GFP-labeled ANXA11 shows that annexin is recruited to the lens structures. In fact, the line profiles across a membrane patch shows a lower density of annexin on the patch surface compared to on the primary membrane outside the patch, supporting that annexin mainly localizes to the lenses in the patch (Fig. 5d,h and i). Imaging of TopFluor-labeled PS lipid shows co-localization between PS and the ANXA11 protein in the lenses (Fig. 5n–q). Lens formation induced by ANXA7 appears identical to lenses induced by ANXA11 (Fig. 5r–u).

**Figure 6:**
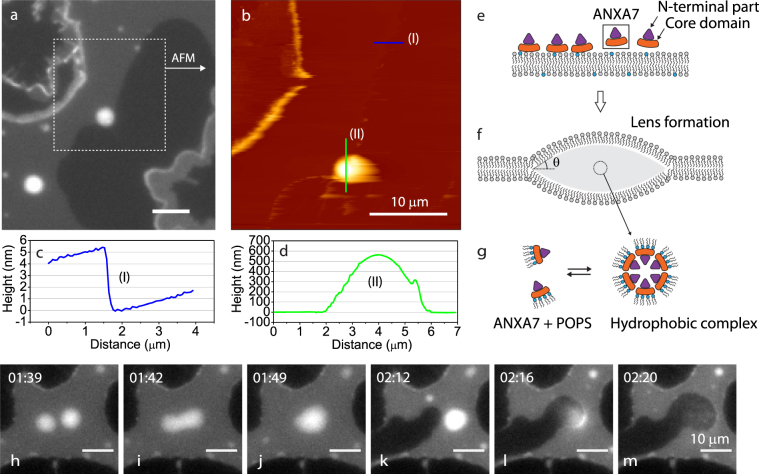
Fluorescence (DiD) image of lens structures formed in a membrane patch after exposure to ANXA7 (**a**). The square indicated in (A) was subsequently scanned with AFM which provides the topography of the lens structure (**b**). Selected line profiles in the AFM topography show the bilayer step height (**c**) and the height variation across a lens (**d**). Conceptual model for lens formation (**e–g**). Model for the formation of hydrophobic reverse micelles containing ANXA7 + POPS that may constitute the interior of a membrane lens (**g**). Fluorescence sequence (DiD) illustrating the fusion of two ANXA7-induced lenses (**h–j**) and the subsequent rupture of the combined lens (**k–m**). Concentrations: ANXA7: 38 nM.

In order to further characterize the lens structures we measured their topography with AFM (Fig. 6). Since the lenses are highly mobile in response to the force from AFM scanning, an immobile lens trapped at the edge of a patch was imaged first by DiD fluorescence (Fig. 6a) and subsequently by AFM (Fig. 6b). The AFM line profile across the patch border shows a step height of 5 nm, corresponding to the expected thickness of the bilayer (Fig. 6c). The lens dimensions obtained from the line profile give a lens radius w = 2 *μ*m and a half-thickness of h = 275 nm (Fig. 6d). The contact angle *θ* of the lens, assuming each half to be a spherical cap can be obtained from the equation:

8 $$\sin \,\theta =\frac{2hw}{{h}^{2}+{w}^{2}}$$

Using the values above, the contact angle of the lens is: *θ* = 15.7° which is similar to values for hydrophobic lenses in lipid bilayers previously measured by Needham *et al*.^48^. The dynamic nature of the lens structures is demonstrated in the time-lapse sequence (Fig. 6h–m). The ANXA7-induced lenses are diffusing in the plane of the patch and merging in a manner resembling the coalescence of drops (Fig. 6i). The growth of lenses by incorporation of lipids and annexin, leads to a loss of membrane area and a contraction of the patch. Interestingly, the retraction of the membrane edge can in some cases lead to the rupture of lenses (Fig. 6k–m). Here the visible loss of content from the lenses and into solution suggests that the lenses contain material localized between the two membrane leaflets.

## Discussion

**Figure 7:**
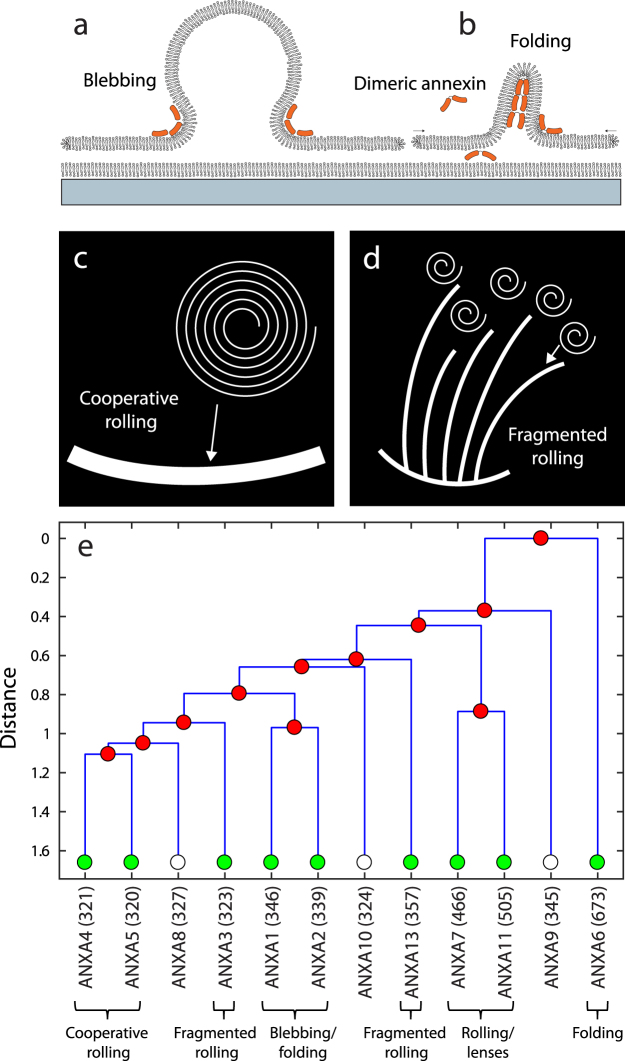
Model for the formation of blebs from membrane patches as induced by single annexins (**a**) and model for the formation of folds generated by the diffusion of dimeric annexin under the membrane patch followed by intercalation between membranes (**b**). Schematic illustration of the difference between cooperative rolling leading to large rolls (**c**) and fragmented rolling leading to multiple thin rolls (**d**). Dendrogram of annexin family members constructed from the respective amino acid sequences (**e**). The x-axis gives the names of annexin members located at the terminal node points plus the number of amino acids for each annexin member (parentheses). The annexins in this study are marked with green and the membrane morphologies induced by each group is indicated (bottom).

Planar membranes with free edges as employed in this study, have revealed distinct morphologies induced by members of the annexin family. This fact highlights that the variation in biological function among the annexins may partly be related to different modes of interaction with the lipid bilayer component of biomembranes. The annexins were grouped according to similarities in the observed membrane structures. ANXA1 and ANXA2 were found to induce blebbing and vesiculation in planar membrane patches, as the only annexins in this study. Since this response is relatively fast, it is probably due to an asymmetric binding of annexin to the top surface of the membrane and subsequent generation of a spontaneous curvature(Fig. 7a). A particular 2D organization of ANXA1 and ANXA2, possibly related to the binding to membrane defects, may promote blebbing rather than rolling for these annexins.

The folding morphology induced by ANXA1 and ANXA6 occurs slower than blebbing and is the main morphological change induced by ANXA6 in addition to perturbation of the membrane edge. The formation of out-of-plane folds suggests a crosslinking of membranes initiated from the underside of the patches. A possible mechanism for this is the slow diffusion of annexin under the patch followed by folding at defect sites (Fig. 7b). By virtue of its two connected core-domains, ANXA6 could be expected to facilitate membrane crosslinking and thereby stabilize folds. ANXA6 was reported by Buzhynskyy *et al*. to crosslink membranes at high Ca^2+^ although the proposed mechanism for crosslinking was not directly mediated by the two core domains^40^. In the case of folding induced by ANXA1, several studies show that ANXA1 and ANXA2 can interact with membranes via the N-terminal region, thereby facilitating crosslinking of adjacent membranes at the monomer level^49^. In the presence of S100 proteins, heterodimerization between two annexins creates a complex with the capacity to crosslink membranes via the calcium binding sites^20,50^.

The cooperative type of membrane rolling is induced by ANXA4, and ANXA5 (Fig. 7c). These annexins have been associated with the formation of trimers on the membrane surface^21,24^ and we showed previously that a trimer-deficient ANXA4 mutant have a strongly reduced rolling capacity^7^. However, the lack of an extended ordered phase of ANXA4 in the AFM image of Fig. 2n suggests that 2D crystallization of annexin is not a requirement for rolling. Rather, results suggest that a particular short-scale molecular arrangement of annexin on the membrane facilitates the formation of large cooperative rolls. The temporal evolution of the rolled membrane area has a sigmoidal profile. If the roll was assumed to have a constant width W one would instead expect the rolling speed to be constant based on equilibration between the rolling force and the hydrodynamic drag force. Therefore, the observed variation in rolling speed mainly reflects an effective variation in the total width of rolls with respect to time. A fit of the rolled area to a logistic function provides a characteristic time scale for the rolling dynamics. Comparison can be made with the characteristic time scale for diffusion of lipids across a patch. With a typical lipid diffusion coefficient in the fluid phase of D $\simeq$ 10^−12^ m^2^/s and a patch dimension of *σ* = 100 *μ*m, the characteristic diffusion time becomes t^*^ = *σ*^2^/4*D* = 2500 s. Since this is ∼1000 times larger than the rolling time constant it gives further support to the interpretation that rolling is caused by a mechanical destabilization induced by spontaneous curvature. Experiments using the annexin core domain (A4ΔN), i.e. ANXA4 with the N-terminal part deleted, was previously presented^7^. These results showed that rolling with a time constant (*τ*) around 2.5 s is induced by A4ΔN, thus corresponding to slower dynamics than ANXA4 and ANXA5.

The rolling morphology induced by annexins has an interesting parallel in the so-called *cochleates* initially discovered in 1975^51,52^. These spiral membrane rolls are spontaneously formed from anionic vesicles upon the addition of divalent cations, typically Ca^2+^, to the outside of vesicles only. Cochleates have attrached attention as promising drug delivery vehicles due to the possibility of incorporating a wide range of molecules into the rolls. Electron microscopy and theoretical modeling have been employed to understand the conditions for formation of cochleates^53,54^. We do not observe rolling without the addition of annexins in our system because both membrane monolayers are exposed equally to Ca^2+^. An increased proportion of PS lipid in combination with assymmetric Ca^2+^ binding might be sufficient to destabilize the membrane patches without annexins. Along this line it was recently shown that assymetric binding of Ca^2+^ to anionic vesicles can generate spontaneous curvature that leads to tubulation^55,56^.

The fragmented pattern of rolls induced by ANXA3 and ANXA13 indicates rolling without cooperativity (Fig. 7d). A possible explanation for this response is that annexins are bound independently without specific in-plane interactions. Although the roll morphology is fragmented in both cases, the detailed pattern is quite different. Previous studies show that the N-terminus of ANXA3 plays a key regulatory role in its membrane binding and membrane permealizing properties^39,57^, which may possibly be connected to the fragmented rolling observed here. According to our model for rolling, it will generally be more energetically favorable to create many rolls with a small diameter compared to fewer and larger rolls.

ANXA7 and ANXA11 are in this study mainly characterized by their unique ability to create fluid and mobile lens structures in the bilayer. Annexin co-localizes with PS lipid in the lenses while annexin is depleted from the outer surface of the membrane. The occasional rupture of lenses and release of their content suggest that lenses encapsulate a hydrophobic material containing annexin and PS lipid localized between the two membrane leaflets. Lenses are predominantly nucleated near the membrane edges and diffuse into the patch, further supporting that their content is enclosed between the monolayers. A possible structure of the lens interior could be a hydrophobic complex of PS and annexin such as reverse micelles or a related geometry (Fig. 6e–g). ANXA7 and ANXA11 both have a large N-terminal region being about 150 amino acids longer than for the other annexins. This region might contribute to the formation of lenses in the presence of PS lipid. It should be noted that fluid lens structures have previously been observed upon the injection of triolein into lipid bilayers^48^ and their budding into lipid droplets were recently studied in GUVs^58^. Triglyceride lenses have been studied by molecular dynamics (MD) simulations of membranes and are generally considered as the first step in the biogenesis of lipid droplets from the endoplasmatic reticulum (ER)^59^.

Further insight into the relationship between the annexin members can be obtained from an analysis of their amino acid sequences. Using bioinformatics algorithms, the full protein sequences were aligned and classified according to their similarity and finally a dendrogram of the human annexins was constructed (Fig. 7e). The full protein sequences were analyzed rather than only the core domain as the N-terminus is considered important for the membrane interactions of annexins. Note that the dendrogram is a simple comparison of annexin sequence similarities and it does not necessarily reflect the evolutionary relationship among the annexins.

Remarkably, the dendrogram reveals a grouping of the annexins which is almost fully in agreement with the grouping of our experimentally observed morphologies in anionic membrane patches. Specifically, the last branch of the tree shows a pairwise grouping of (ANXA1, ANXA2), (ANXA4, ANXA5) and (ANXA7, ANXA11) corresponding to the pairwise similarities in our membrane experiments which display distinct morphologies. ANXA3 and ANXA13 are not closely related in the dendrogram, suggesting that fragmented rolling can be induced by structurally different annexins that both fail to induce cooperativity when generating membrane curvature. ANXA6 which induces folding in addition to ANXA1, comes out highly separated from the other members due to the presence of two core domains. The core domain of the annexins is responsible for the primary binding to membranes and is relatively conserved. The main variation is found in the N-terminal region which may influence the protein-protein interaction between annexins. This in turn, could influence membrane morphologies through different spatial configurations of annexins bound to lipids. In conclusion, the ability to induce membrane curvature seems to be a shared characteristic feature for many members of the annexin protein family, which possibly play an important role for annexin function.

## Methods

### Production of recombinant proteins

Recombinant proteins were produced from PCR amplified ANXA cDNA subcloned into the bacterial expression vector pETM11-SUMO3 with or without C-terminally tagged superfolder GFP (sfGFP)^60^ and N-terminally tagged with a hexahistidine and SUMO3 domain. Lact-C2-GFP was produced as described in^61,62^. ANXA4-Ca3mut only having one Ca^2+^ binding site was generated by PCR-based site-directed mutagenesis by replacing amino 71E, 143E and 227E to A^63^. Proteins were expressed overnight at 16 °C in competent Escherichia coli BL21 (DE3) cells using Isopropyl *β*-D-1-thiogalactopyranoside (IPTG). Next cells were lysed (50 mM TrisHCl pH 7.5, 500 mM NaCl, 20 mM imidazole, 0.5 mM DTT, complete EDTA-free protease inhibitor cocktail (Roche)) in the presence of 10% 10 × Bugbuster Extraction Reagent (Novagen) and benzonase (Sigma-Aldrich). The lysate was centrifuged at 5.100 g for 45 min at 4 °C and the supernatant was filtered (0.45 *μ*m). Soluble proteins were purified by Immobilized Metal-Affinity Chromatography (IMAC) using nickel-nitrilotriacetic acid (Ni-NTA) resins (Qiagen) followed by wash (50 mM TrisHCl pH 7.5, 250 mM NaCl) and elution (50 mM TrisHCl pH 7.5, 250 mM NaCl, 300 mM imidazole). The N-terminal hexahistidine and SUMO3 domain-tag were cleaved off by SenP2 protease (molar ratio 200:1) while dialyzing (50 mM TrisHCl pH 7.5, 250 mM NaCl) overnight at 4 °C. The tag-cleaved protein with DTT (5 mM) was separated via Fast Protein Liquid Chromatography (FPLC) on a Superdex 200 size-exclusion chromatography column (GE Healthcare Life Science), and protein fractions were pooled and stored at −80 °C. Technical challenges in the production of ANXA8, ANXA9 and ANXA10 forced us to exclude these proteins in the study and direct our focus to the remaining annexin family members.

### Preparation of multilayered spincoated supported membranes

Mica substrates (Plano GmbH) were glued to glass coverslips using a silicone elastomer (MED-6215, Nusil Technology) and cleaved immediately before use. Dry spin-coated lipid films of phosphatidylcholine (POPC; 1-hexadecanoyl-2–(9Z-octadecenoyl)-sn-glycero-3-phosphocholine) and phosphatidylserine (POPS; 1-hexadecanoyl-2–(9Z-octadecenoyl)-sn-glycero-3-phospho-L-serine) on mica substrates were prepared from a stock solution containing 10 mM total lipid (POPC, POPS, 9:1 molar ratio) and 0.5% DiD-C18 probe (Thermo, Invitrogen). A 40 *μ*L droplet of the lipid stock was applied to the mica, spun on a spincoater (KW-4A, Chemat Technology) at 3000 rpm for 40 s and placed under vacuum in a desiccator for 10–12 hours to ensure evaporation of the solvent. The spincoated lipid film was hydrated in 10 mM TRIS buffer (2-Amino-2-(hydroxymethyl)propane-1,3-diol), 140 mM NaCl, 2 mM Ca^2+^, pH = 7.4 at 55 °C for 2 hours. Then the sample was gently flushed with 55 °C buffer and buffer exchanged >10 times to prepare defined secondary bilayer patches resting on a continuous primary membrane. The hydrated membrane patches where cooled to 22 °C and equilibrated for 1–2 hours before further experiments. The response of bilayer patches to addition of annexin was monitored at 22 °C with time-lapse epi-fluorescence microscopy (DiD).

### Epi-fluorescence microscopy of response to annexin

A Nikon TE2000 inverted microscope with 40× long working distance objective (Nikon ELWD, Plan Fluor, NA = 0.6) was used for epi-fluorescence observations. Fluorescence excitation was performed with a switchable Xenon lamp (PolychromeV, Till Photonics GmbH, Grafeling, Germany) and a dual wavelength filter cube for imaging at 640 nm (DiD) and 488 nm (GFP). Images were recorded with an emccd camera (Sensicam em, 1004 × 1002 pixels, PCO-imaging, Kelheim, Germany) and operated with Live Aquisition software (FEI GmbH). Epi-fluorescence images were analyzed with MATLAB (The Mathworks) and ImageJ (National Institute of Health). Annexin in an absolute amount of 100 pmole was added to the fluid cell from a concentrated stock with a known concentration of typically 1 mg/mL, and the sample imaged at 3–10 frames per second, depending on the response speed. Each experiment was repeated a minimum of n = 3 times except ANXA6 and Lact-C2-GFP (n = 2). GFP-labeled annexins were imaged simultaneously to DiD by wavelength switching of the excitation.

### AFM imaging

Atomic force microscopy was performed using a JPK Nanowizard I AFM system (JPK Instruments AG) operated in contact mode. The AFM is mounted on the Nikon TE2000 fluorescence microscope described above. Cantilevers where of the type MSCT, lever C, with spring constant = 0.01 N/m (Bruker Corporation). Supported membrane samples were imaged in buffer with the smallest possible contact force to minimize potential membrane deformations. Processing of AFM images was done with SPM Data Processing (JPK) or Scanning Probe Image Processor (SPIP, Image Metrology A/S).

### Image analysis

Time-lapse sequences of membrane rolling were analyzed in MATLAB (The Mathworks). The incremental rolled membrane area was determined by subtraction of subsequent frames in the sequence followed by binarization with a cutoff. The total rolled area as function of time was fitted by a logistic (sigmoid) function with a time constant *τ*, which provides a characteristic time scale for the rolling process. The theoretical model for rolling energetics was implemented and plotted in MATLAB (The Mathworks).

### Protein sequence analysis

Sequence analysis of annexins (human) was performed in MATLAB using the Bioinformatics toolbox. Amino acid (AA) sequences for were obtained from the Protein database^64^. The following sequence codes were used: ANXA1: NP_000691, ANXA2: AAH68065, ANXA3: NP_005130, ANXA4: EAW99844, ANXA5: NP_001145, ANXA6: AAH17046, ANXA7: AAH02632, ANXA8: AAH73755, ANXA9: NP_003559, ANXA10: NP_009124, ANXA11: CAB94997, ANXA13 (isoform b): NP_001003954.

First, multiple alignment of the full amino acid sequences for annexins was performed using the Blocks Substitution Matrix (BLOSUM). The alignment algorithm aligns the annexin core domain between annexin members. In the case of ANXA6, the second (N-terminal) core domain is aligned. Secondly, the pairwise distance between aligned sequences was calculated using the Jukes-Cantor method and finally a dendrogram was constructed using the Unweighted Pair Group Method Average (UWPGMA). For comparison, the Weighted Pair Group Method Average (WPGMA) method was also applied and this yielded a dendrogram with a structure identical to the UWPGMA dendrogram. See Supplementary Fig. S10 for the MATLAB code used to produce Fig. 7e and Supplementary Fig. S11 for the WPGMA dendrogram.

### Data availability

The datasets generated during and analysed during the current study are available from the corresponding author on reasonable request.

## Electronic supplementary material

Supplementary information
