# Supplementary material for: Annexins induce curvature on free-edge membranes displaying distinct morphologies
Source: Sci Rep. 2018 Jul 9;8:10309. doi: 10.1038/s41598-018-28481-z (PMC6037701; doi:10.1038/s41598-018-28481-z)
Supplement: Supplementary file 1 — Supplementary information [file 41598_2018_28481_MOESM1_ESM.pdf]

Supplementary information: Annexins induce curvature on free-edge membranes displaying distinct morphologies

Theresa Louise Boye[4], Jonas Camillus Jeppesen[1,2], Kenji Maeda[4], Weria Pezeshkian[1,2], Vita Solovyeva[1,3], Jesper Nylandsted[4,5], and Adam Cohen Simonsen[1,2,*]

[*]Corresponding author: adam@memphys.sdu.dk

[1]University of Southern Denmark (SDU), Campusvej 55, DK-5230 Odense M, Denmark

[2]Department of Physics Chemistry and Pharmacy

[3]Department of Biochemistry and Molecular Biology

[4]Membrane Integrity Group, Unit for Cell Death and Metabolism, Danish Cancer Society Research Center, Strandboulevarden 49, DK-2100 Copenhagen, Denmark.

[5]Department of Cellular and Molecular Medicine, Faculty of Health Sciences, University of Copenhagen, DK-2200 Copenhagen N, Denmark.

1 Model for membrane rolling on a support with adhesion

1.1 Condition for stability of a supported membrane

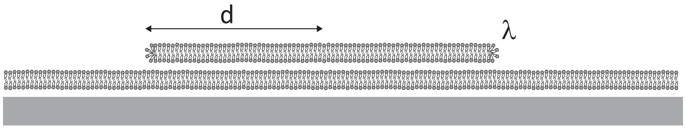

Figure S1: Stability of a supported membrane patch of radius d. The free membrane edge has a line tension λ.

We consider a membrane placed on top of a primary supported membrane. The top membrane is mechanically stable if its attractive interaction energy with the primary supported membrane is larger than the line tension energy of the membrane edges. The adhesion energy is proportional to the membrane area while the line tension is proportional to its total perimeter. Considering initially a disk-shaped membrane, there is a critical radius $d_0 = 2\lambda/w_{\rm ad}$ below which the bilayer is unstable and will transform to a vesicle. Here λ is the line tension of the free membrane edge and $w_{\rm ad}$ is the adhesion energy per area. For $d > d_0$, the membrane can be stable and remain flat. However, upon binding of curvature inducing proteins (e.g. annexins) there is an curvature energy penalty in the flat configuration due to a spontaneous curvature (c_0) induced by the proteins bound to the membrane. If this energy penalty is sufficiently large, the top membrane may start to curve, even for large areas where $d \gg d_0$.

1.2 Initiation of membrane rolling

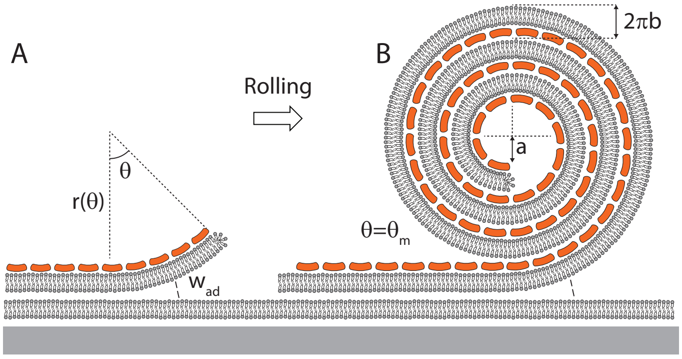

Figure S2: Illustration of the roll-up of a secondary planar membrane on a primary supported membrane. Binding of curvature inducing proteins to the membrane surface induces a spontaneous curvature c_0. Initiation of rolling (equation 4) occurs by detachment of the free membrane edge from the support (A). Rolling is energetically favored until the roll reaches a maximal radius r_m or equivalently, if the rolled angle θ reaches θ_m (B).

We consider the rolling of a planar membrane on a support surface consisting of a primary membrane and a solid material underneath. In the initial state, the membrane is flat and is terminated by a linear free edge. Upon binding of a protein (e.g. annexins) to the membrane surface the effect of the protein is modeled as the induction of a spontaneous curvature (c_0) of the combined membrane/protein sheet. We will determine the energy difference between the initial flat configuration and a final state where the membrane is rolled. The energy of the initial (flat) state is:

$$E_0 = E_{0A} + E_{\rm ad} = A(\frac{k_c}{2}c_0^2 - w_{\rm ad}) \tag{1}$$

The energy in the final (rolled) state is:

$$E_1 = \int_A \left[\frac{k_c}{2}(\bar{c} - c_0)^2\right] dA \tag{2}$$

where k_c is the *mean curvature elastic modulus*[1]. In the case of a linear roll, the mean curvature $\overline{c} = \frac{1}{R_1} + \frac{1}{R_2}$ reduces to $\overline{c} = \frac{1}{R(s)}$ where $R(s)$ is the local radius of curvature in the roll at arc length s. The energy change for a roll of width W from the flat to the rolled state is:

$$\Delta E = E_1 - E_0 = W \int_0^{s_{\max}} \left[\frac{k_c}{2} \left(\frac{1}{R(s)} - c_0 \right)^2 - \frac{k_c}{2} c_0^2 + w_{\text{ad}} \right] ds \tag{3}$$

From equation (3) it can be concluded that ΔE is minimal when $\frac{1}{R(s)} = c_0$. Rolling is energetically favored when $\Delta E < 0$ or when:

$$\frac{k_c}{2} c_0^2 > w_{\text{ad}} \tag{4}$$

Equation (4) provides a condition for the initiation of rolling.

1.3 Rolling

Once the barrier in equation (4) has been overcome, the membrane can roll and separate from the bilayer continuously. However, during rolling the curvature radius $R(s)$ increases and eventually the adhesion energy will overcome the gain in curvature elastic energy and rolling stops. Experimentally we find that rolling proceeds to distances of at least 100 μm. Below we investigate the energetics of the rolling process and determine the rolled distance L in terms of the system parameters k_c, c_0 and w_{ad}.
First we note that equation (3) simplifies to:

$$\Delta E = W \int_0^{s_{\max}} \left[\frac{k_c}{2} \left(\frac{1}{R(s)^2} - \frac{2c_0}{R(s)} \right) + w_{\text{ad}} \right] ds \tag{5}$$

To model the rolling process, we assume that the roll is shaped as an archimedean spiral defined by: $r(\theta) = a + b\theta$. Here $r(\theta)$ is the radius of the spiral at the rolling angle θ, a is the radius of the inner roll and $2\pi b$ is the repeat distance between roll layers. In the following, the parameter a will be determined by energy minimization while b is estimated from experiments. A significant simplification is obtained if the curvature radius is approximated by the radius of the spiral: $R \approx r$. We discuss the validity of this approximation in the subsequent section. We also note that $ds \approx r d\theta = \frac{r}{b} dr$.
With these simplifications, equation (5) becomes:

$$\Delta E = \frac{W k_c}{b} \int_a^{a+b\theta} \left(\frac{1}{2r} - c_0 + \frac{w_{\text{ad}}}{k_c} r \right) dr \tag{6}$$

After integration, equation (6) becomes:

$$\Delta E = \frac{W k_c}{b} \left[\frac{1}{2} \ln \left(1 + \frac{b}{a} \theta \right) + \left(\frac{w_{\text{ad}}}{k_c} ab - c_0 b \right) \theta + \frac{1}{2} \frac{w_{\text{ad}}}{k_c} b^2 \theta^2 \right] \tag{7}$$

The variables θ and a are determined by minimization of the energy change ΔE:

$$\frac{\partial \Delta E}{\partial \theta} |_{\theta = \theta_m} = 0, \qquad \frac{\partial \Delta E}{\partial a} |_{a = a^*} = 0 \tag{8}$$

Here θ_m is the maximal rolling angle corresponding to the angle when rolling stops. Corresponding to this is the maximum (final) radius of the roll: $r_m = a + b\theta_m$. By inserting ΔE from equation (7) we arrive at the following expressions for the parameters in the final state of the roll:

Maximum roll radius r_m:

$$r_m = \frac{c_0 + \sqrt{c_0^2 - 2 \frac{w_{\text{ad}}}{k_c}}}{2 \frac{w_{\text{ad}}}{k_c}} \tag{9}$$

The inner radius a:

$$a = \left[2 \frac{w_{\text{ad}}}{k_c} r_m \right]^{-1} \tag{10}$$

The maximal rolling angle θ_m:

$$\theta_m = \frac{r_m - a}{b} \tag{11}$$

Rolled distance L:

$$L = a\theta_m + \frac{b\theta_m^2}{2} \tag{12}$$

The slope b of the archimedean spiral can be found as:

$$b = \frac{r_m^2 - a^2}{2L} \tag{13}$$

From which the layer spacing $2\pi b$ is easily found from experimentally measured values of L and r_m.

1.4 Rolling model without approximated curvature radius

We now investigate the rolling model in the case where the curvature radius R is described exactly and not approximated by r. Given the expression $r(\theta) = a + b\theta$ for the spiral, the radius of curvature is given exactly as:

$$R = \frac{(r^2 + r_\theta^2)^{3/2}}{|r^2 + 2r_\theta^2 - r r_{\theta\theta}|} = \frac{(r^2 + b^2)^{3/2}}{r^2 + 2b^2} \tag{14}$$

where $r_\theta = \frac{dr}{d\theta}$ and $r_{\theta\theta} = \frac{d^2r}{d\theta^2}$. The arc length s is given as:

$$ds = \sqrt{r^2 + r_\theta^2}\ d\theta = \sqrt{r^2 + b^2}\ d\theta = \frac{1}{b}\sqrt{r^2 + b^2}\ dr \tag{15}$$

Inserting equation (14) and (15) into equation (5) we obtain:

$$\Delta E = \frac{W k_c}{b} \int_a^{a+b\theta} \left[\frac{(r^2 + 2b^2)^2}{2(r^2 + b^2)^{5/2}} - c_0 \frac{(r^2 + 2b^2)}{(r^2 + b^2)} + \frac{w_{\mathrm{ad}}}{k_c}(r^2 + b^2)^{1/2} \right] dr \tag{16}$$

Next we need to find the minimum of ΔE according to equations (8). Here we take advantage of Leibnitz rule:

$$\frac{d}{dx}\left[\int_{f_1(x)}^{f_2(x)} g(t)dt\right] = g(f_2(x))f_2'(x) - g(f_1(x))f_1'(x) \tag{17}$$

Minimization of ΔE in equation (16) leads to the following polynomium:

$$2\frac{w_{\mathrm{ad}}}{k_c}x^6 - 2c_0x^5 + x^4 - 2c_0b^2x^3 + 2b^2x^2 + b^4 = 0 \tag{18}$$

with the roots x_1 and x_2 given as:

$$x_1^2 = a^2 + b^2, \qquad x_2^2 = (a + b\theta_m)^2 + b^2 \tag{19}$$

Equation (18) is solved numerically and the value of a and θ_m found from equations (19). The rolled distance L is determined by integration of ds:

$$L = \int ds = \frac{1}{b}\int_a^{r_m} \sqrt{r^2 + b^2}\ dr \tag{20}$$

$$= \frac{1}{b}\left[\frac{r_m}{2}\sqrt{r_m^2 + b^2} + \frac{b^2}{2}\ln(r_m + \sqrt{r_m^2 + b^2}) - \frac{a}{2}\sqrt{a^2 + b^2} - \frac{b^2}{2}\ln(a + \sqrt{a^2 + b^2})\right] \tag{21}$$

1.5 Results

Next we evaluate the rolled distance and compare the results of the two models described above. We estimate values for the parameters: k_c, c_0, w_{ad} and b. Precise values corresponding to our experimental system (POPC, POPS 9:1 or cellular membranes) are not available in the literature and we therefore use the following order-of-magnitude estimates based on similar lipid systems:

- k_c=4.0·10^{-20} J. From Marsh[2], table II.10.4.1, page 474. Reported values for POPC are from 2.5-8.5·10^{-20} J.
- c_0=0.033 nm^{-1}. Based on computational modeling of Shiga toxin by Pezeshkian et. al. [3]. Recent results on Cholera toxin yield a spontaneous curvature of c_0=0.028 nm^{-1}[4].
- b=12nm/2π. This is based on AFM measurements of roll diameters for annexin A4[5].
- w_{ad} will cover a range of relevant values. Literature values for neutral SOPC bilayers in 0.1 M PBS report a value of $w_{\text{ad}} \simeq 1.0 \cdot 10^{-5}$ J/m^2 [6].

A comparison of the rolled distance L versus w_{ad}/k_c for the two models described above is shown in Figure S3. Values of the system parameters are indicated above. The two models are indistinguishable on a double-logarithmic scale and therefore the approximate model gives a reasonable estimation of the rolled length L.

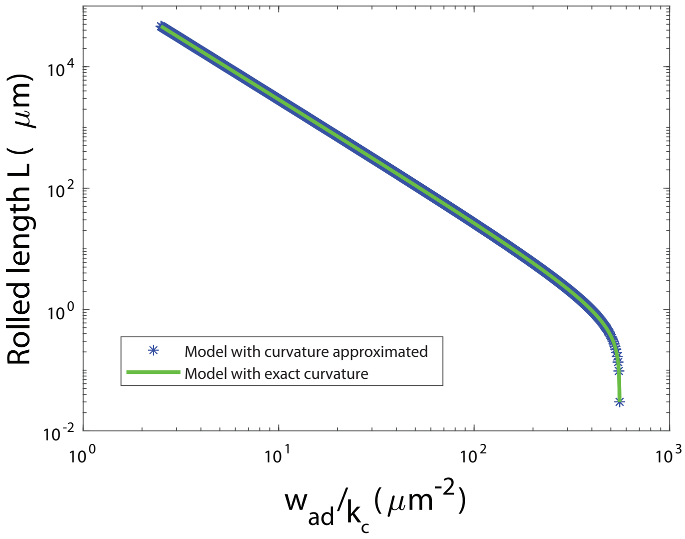

Figure S3: Comparison of the rolled length L versus w_{ad}/k_c obtained with the approximated curvature radius (equation 12) and the exact curvature (equation 21). The mean curvature modulus was fixed at k_c=4.0·10^{-20} J.

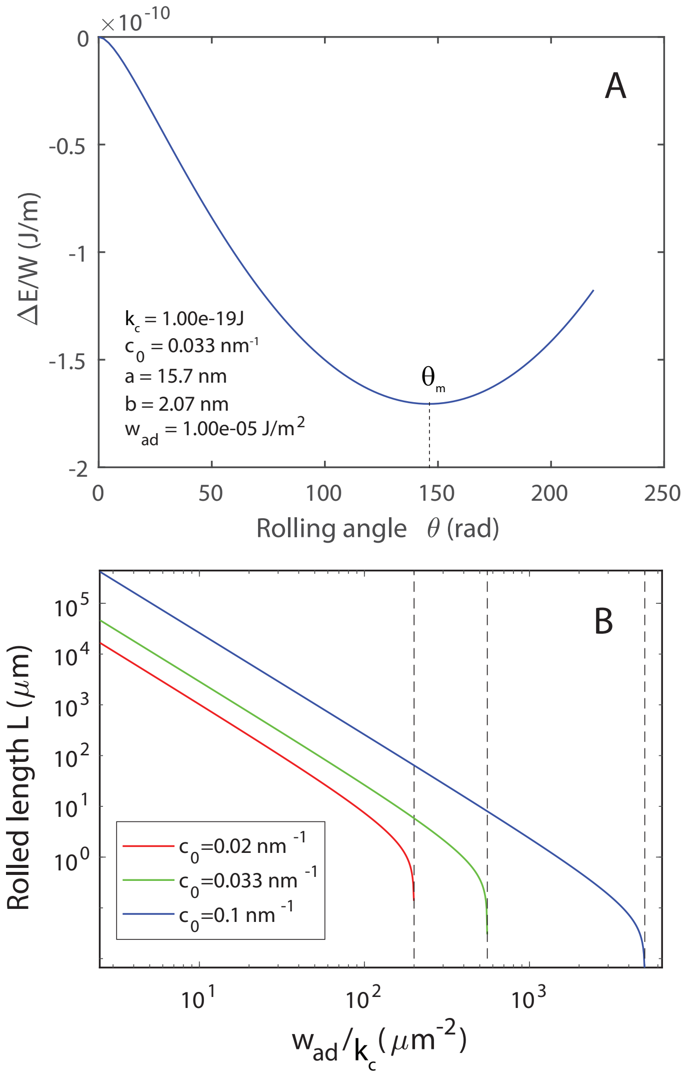

Figure S4: The energy difference between the flat (initial) and the rolled (final) state of a membrane according to equation (7) (A). The system parameters used are indicated in (A). The stable roll configuration corresponds to the energy minimum as described by the rolling angle $\theta = \theta_m$. The rolled length L versus w_{ad}/k_c obtained with the approximated curvature radius (equation 12) (B). The mean curvature modulus was fixed at k_c=4.0·10^{-20} J. Curves for 3 representative value of c_0 are shown and for each curve, the dashed vertical line represents the critical value of the adhesion energy for initiating rolling (equation 4).

2 Controls and extended datasets for annexin experiments

Below is a series of extended data for the annexins interacting with membrane patches. The membrane composition is in all cases: POPC,POPS (90%:10%).

2.1 Negative controls: Absence of Ca^{2+}, ANXA4-Ca3mut, Lact-C2-GFP

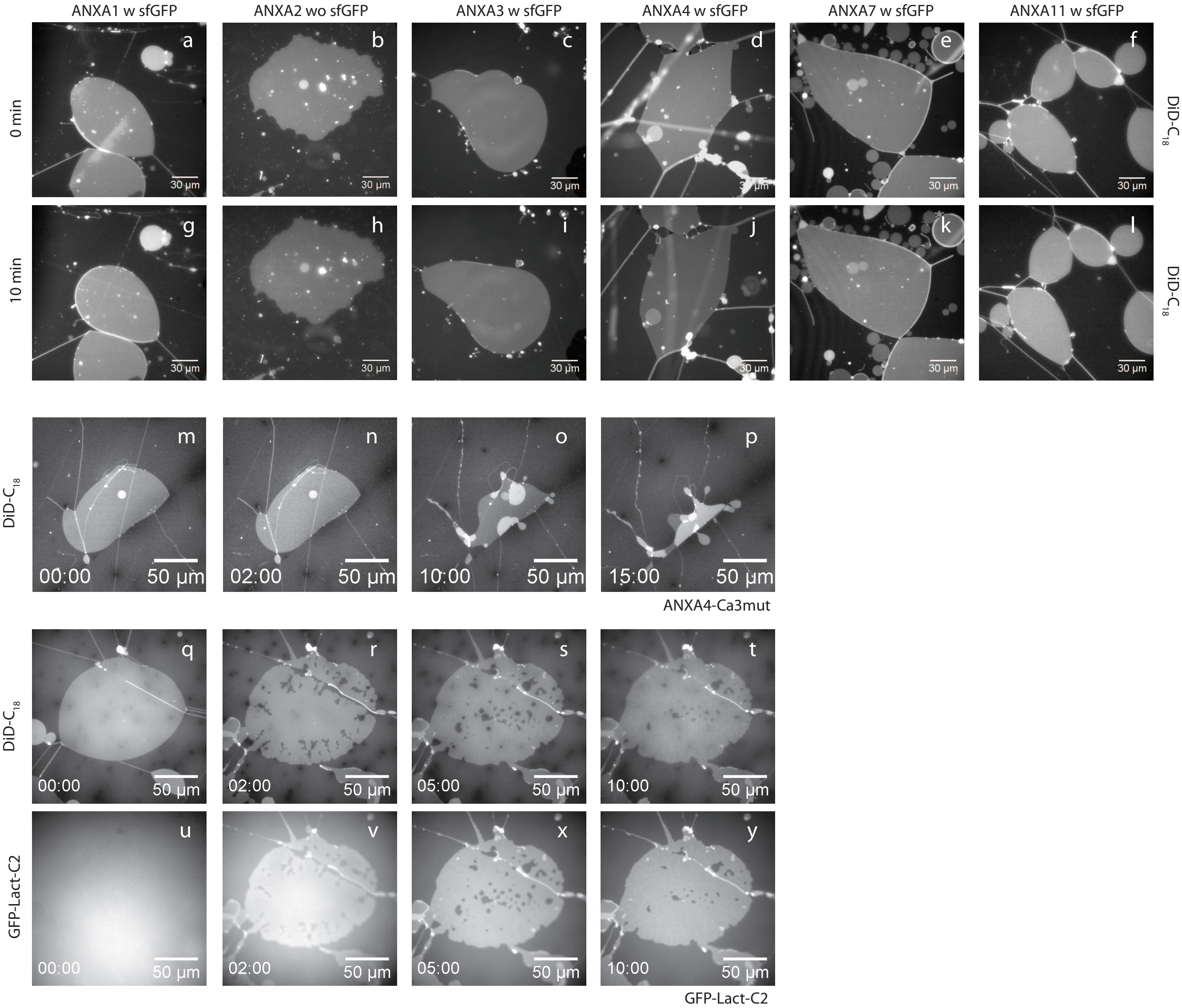

Figure S5: Negative control experiments for membrane patches exposed to annexins in the absence of Ca^{2+}. Frames (a-f) show the membrane patches immediately before addition of annexin while frames (g-l) show the same patches 10 min after exposure to 13 nM annexin with the type indicated above frames. There is no response of the membrane patches to annexin except for a weak physical relaxation of the membrane shape in some cases (e.g. c to i) which is also observed without annexin. For comparison, the time point 10 min shows a strong response to all tested annexin types in the presence of 2 mM Ca^{2+}. Control experiment with ANXA4-Ca3mut (m-p), a mutation of ANXA4 where 3 out of 4 Ca^{2+}-binding sites are passivated. Binding does not induce rolling, but instead leads to a slow vesiculation from the patch within 10-15 min. Control experiment with the PS-binding domain Lact-C2-GFP from lactadherin (q-y)[7]. In this case binding is observed in the GFP channel and does not produce rolling. However, small holes in the membrane patch are generated by Lact-C2-GFP and these are partly closed again after 10 min. Concentrations: ANXA4-Ca3mut: 43 nM, Lact-C2-GFP: 81 nM.

2.2 Extended dataset for ANXA1 and ANXA2

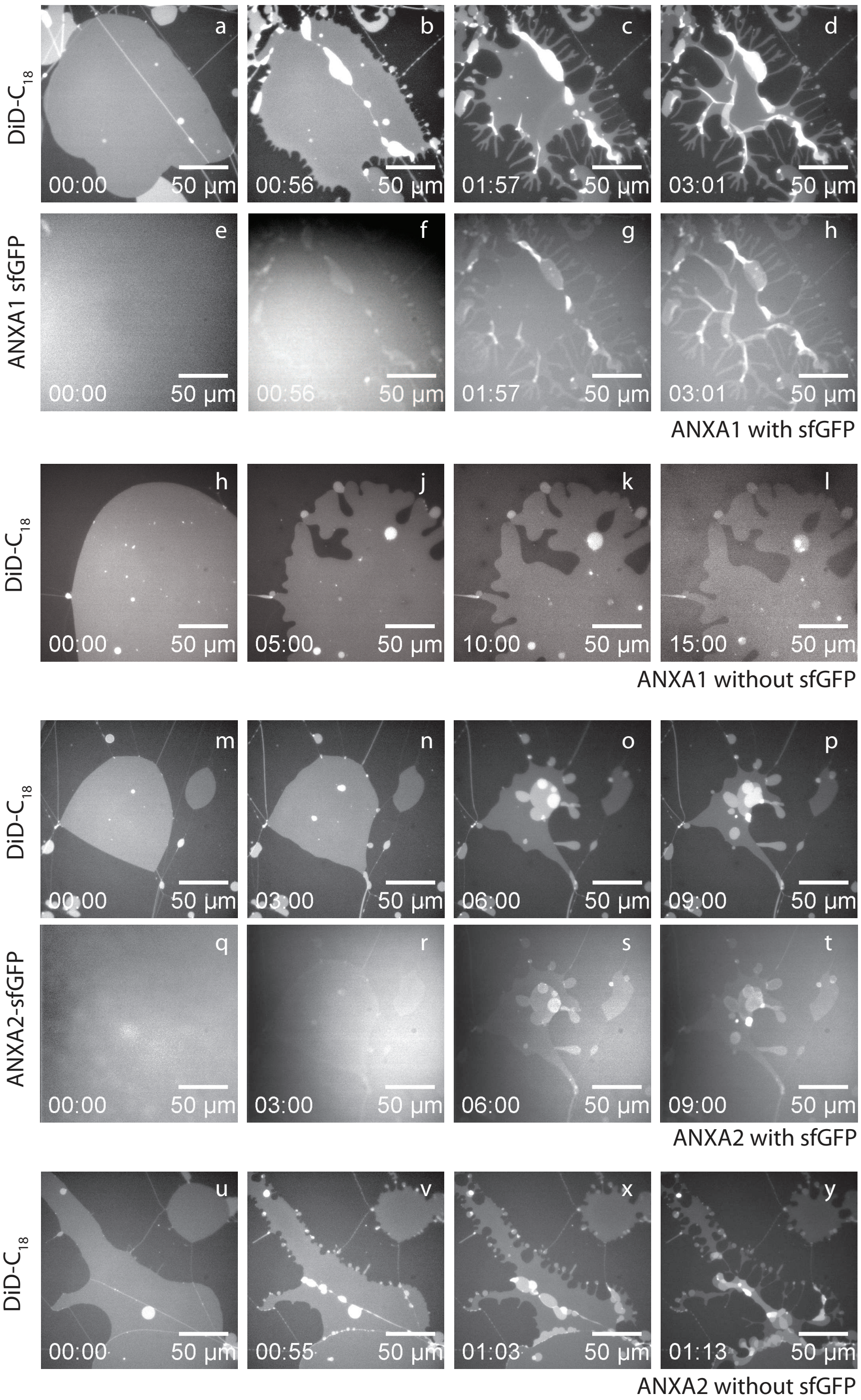

Figure S6: Extended dataset for membrane patches exposed to ANXA1 and ANXA2. Frames (h-l) show data for ANXA1 without sfGFP and frames (u-y) show data for ANXA2 without sfGFP. In addition, frames (q-t) show the GFP channel (488 nm) for ANXA2 with sfGFP. The extended data sets without sfGFP confirm that the GFP tag (see main article) has no visible influence on the type of morphological changes induced in the membrane patches. The data for ANXA2 w sfGFP show that ANXA2 is accumulated in the bleb structures corresponding to what is observed for ANXA1. Concentrations: ANXA1-sfGFP: 46 nM, ANXA1: 48 nM, ANXA2-sfGFP: 58 nM, ANXA2: 40 nM.

2.3 Extended dataset for ANXA6

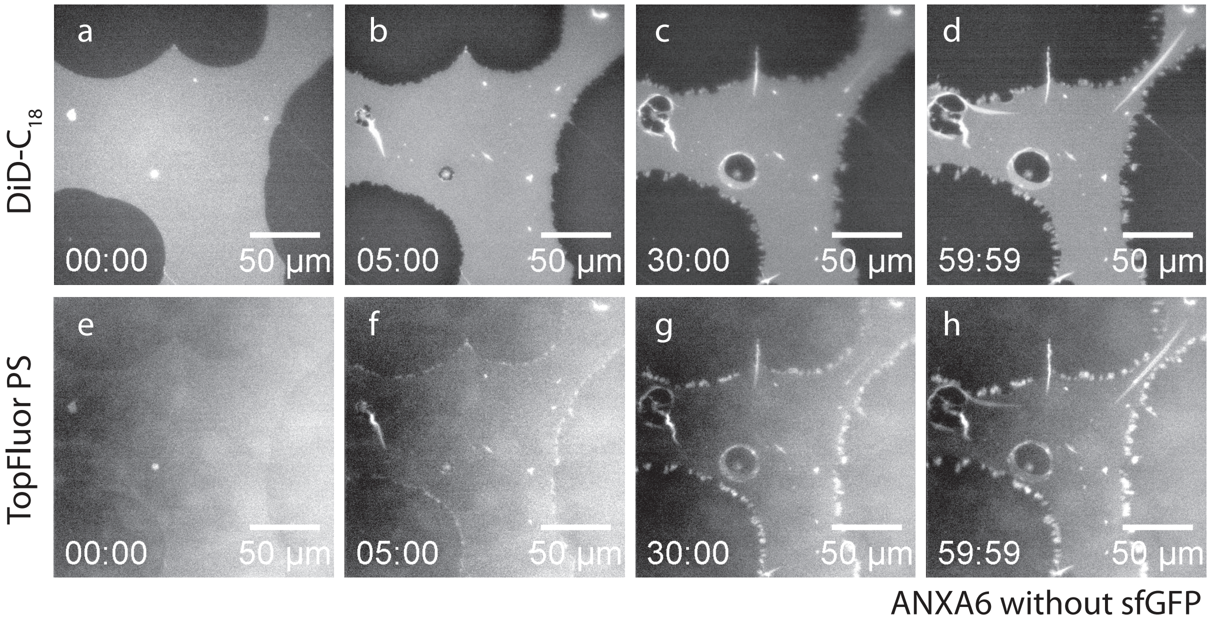

Figure S7: Dataset for ANXA6 extended with images of TopFluorPS (488 nm, frames e-h). The data shows that the PS lipid is recruited to the membrane edges upon addition of ANXA6 and to the folding structures emerging from the membrane patch. Concentrations: ANXA6: 50 nM.

2.4 Extended dataset for ANXA7

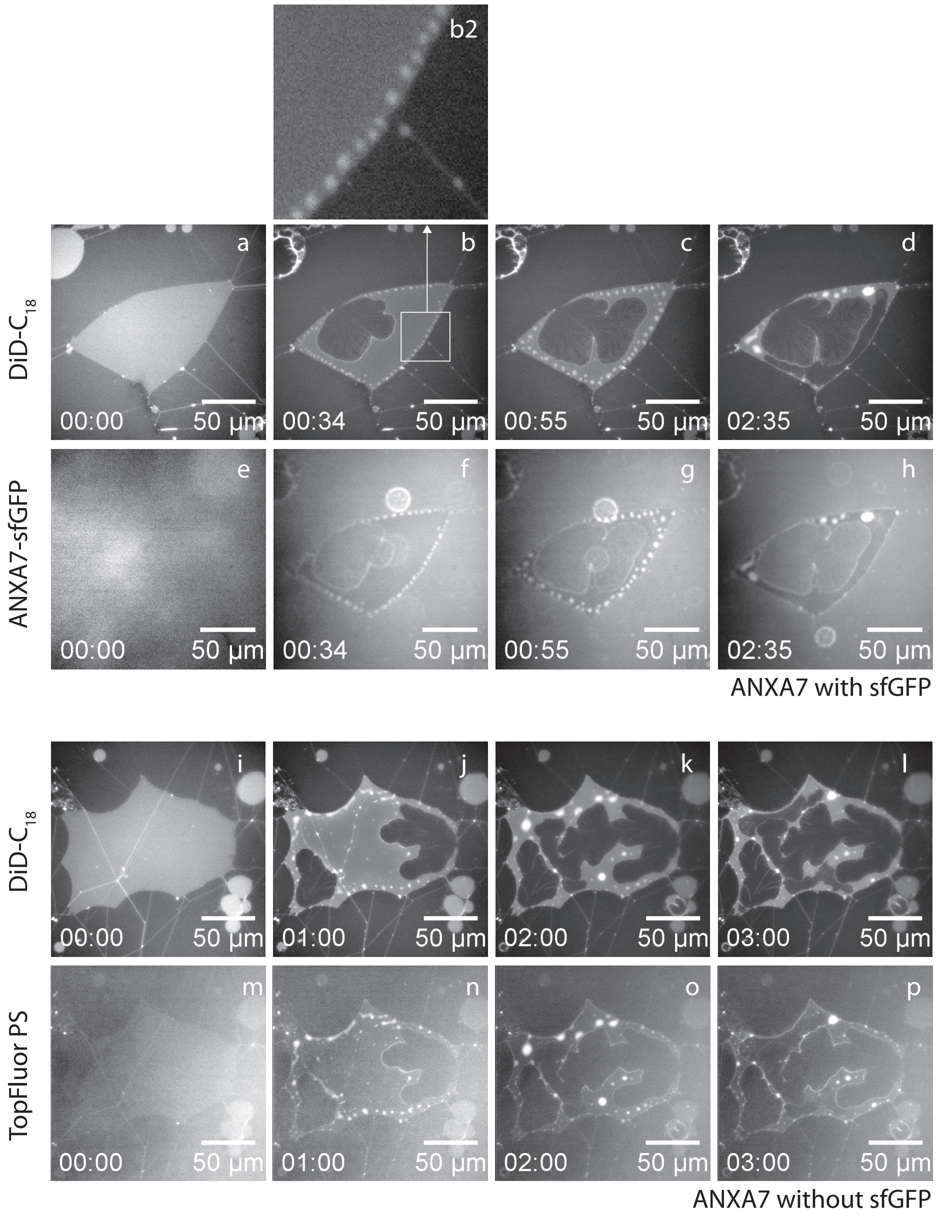

Figure S8: Extended data for membrane patches exposed to ANXA7. Frames (a-d) and (e-h) show the response of a membrane patch upon exposure to ANXA7 with sfGFP in the DiD and the sfGFP channels respectively. Frames (i-l) and (m-p) show the response to ANXA7 without sfGFP and confirm that the GFP tag has no visible influence on the morphological changes induced in the membrane patch. The magnification in (b2) demonstrates that lenses are nucleated uniformly near the membrane edge. Frames (e-h) and (m-n)show that ANXA7 and PS lipid are both accumulated in the lens structures. Overall, the morphological changes induced by ANXA7 are fully equivalent to the observations made for ANXA11. Concentrations: ANXA7-sfGFP: 23 nM, ANXA7: 38 nM.

2.5 Extended dataset for ANXA3 and ANXA13

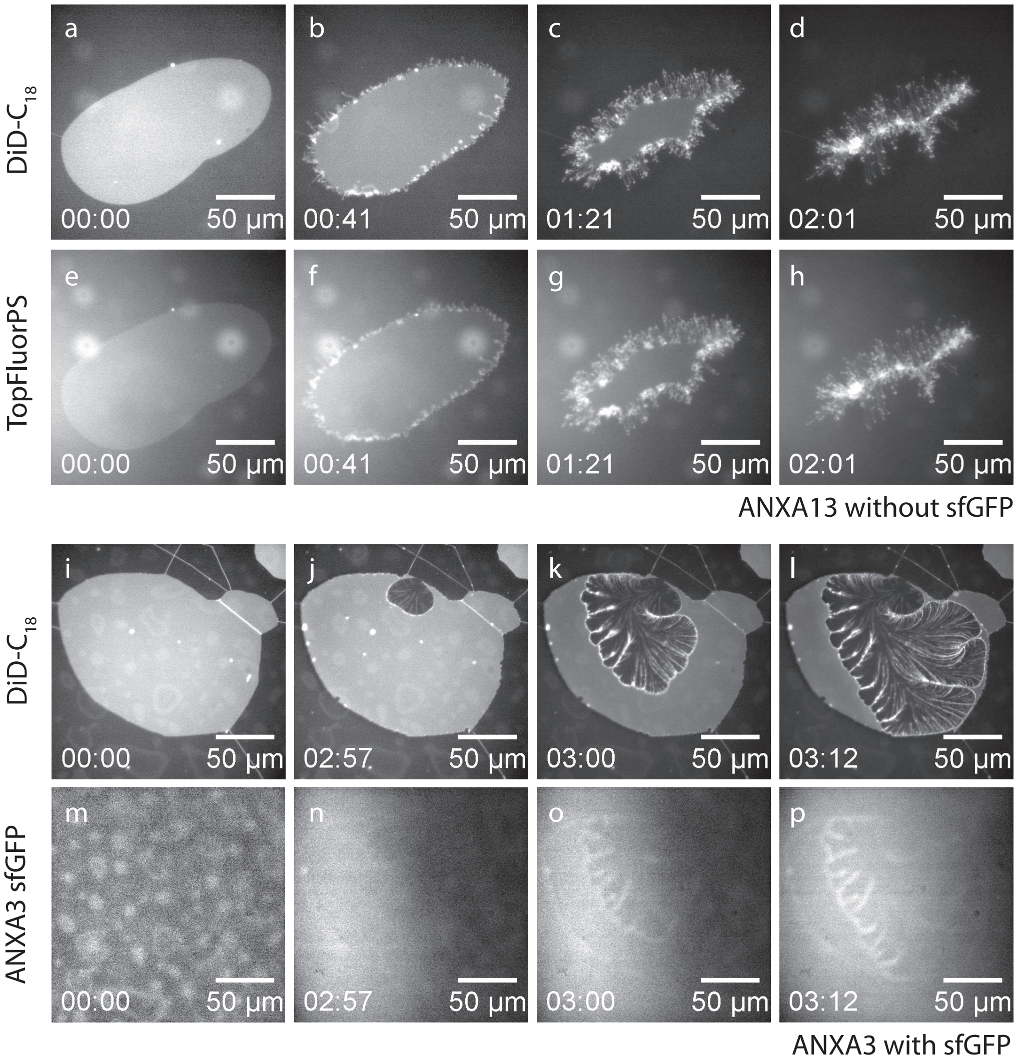

Figure S9: Data for ANXA13 and ANXA3 extended with TopFluorPS images for ANXA13 (e-h) and sfGFP images for ANXA3 (m-p). The data show that annexin and PS lipid is accumulated in the fragmented roll structures. Concentrations: ANXA13: 53 nM, ANXA3-sfGFP: 49 nM.

3 MATLAB code for dendrograms

```
% MATLAB code for construction of dendrogram for human annexins
% Full code used to generate Fig. 7e.
% Codes are obtained from https://www.ncbi.nlm.nih.gov/protein/
clear all;
Codes = {'ANXA1' 'NP_000691';
        'ANXA2' 'AAH68065';
        'ANXA3' 'NP_005130';
        'ANXA4' 'EAW99844';
        'ANXA5' 'NP_001145';
        'ANXA6' 'AAH17046';
        'ANXA7' 'AAH02632';
        'ANXA8' 'AAH73755';
        'ANXA9' 'NP_003559';
        'ANXA10' 'NP_009124';
        'ANXA11' 'CAB94997';
        'ANXA13' 'NP_001003954';
        };
% Downloading protein (AA) sequences and put them into 'seqs' structure:
for ind = 1:length(Codes)
    AnnexinData(ind) = getgenpept(Codes{ind,2}); % retrieve full info from databank
    seqs(ind).Sequence = AnnexinData(ind).Sequence;
    seqs(ind).Header = [Codes{ind,1} ' (' num2str(AnnexinData(ind).LocusSequenceLength) ')']; % place header names in the seqs structure array
end
%% alignment and tree generation
SeqsMultiAligned = multialign(seqs); % align annexin sequences
distances = seqpdist(SeqsMultiAligned,'Method','Jukes-Cantor'); % Calculating distances between aligned sequences
tree = seqlinkage(distances,'average',SeqsMultiAligned); % make dendrogram with UWPGMA method
%tree = seqlinkage(distances,'weighted',SeqsMultiAligned); % make dendrogram with WPGMA method
%% plot dendrogram
h = plot(tree,'orient','top');
ylabel('Distance','FontSize',8)
set(h.terminalNodeLabels,'Rotation',90,'FontSize',8)
set(h.BranchDots,'MarkerFaceColor','r','MarkerSize',9);
set(h.axes,'LineWidth',1,'FontSize',8);
set(h.BranchLines,'LineWidth',1,'Color','b');
set(h.LeafDots,'Marker','o','MarkerFaceColor','k','MarkerFaceColor','w','MarkerSize',9)
```

Figure S10: MATLAB code for construction of dendrogram for human annexins. Full code used to generate Fig. 7e and Fig. S11

3.1 Supplementary dendrogram: WPGMA method

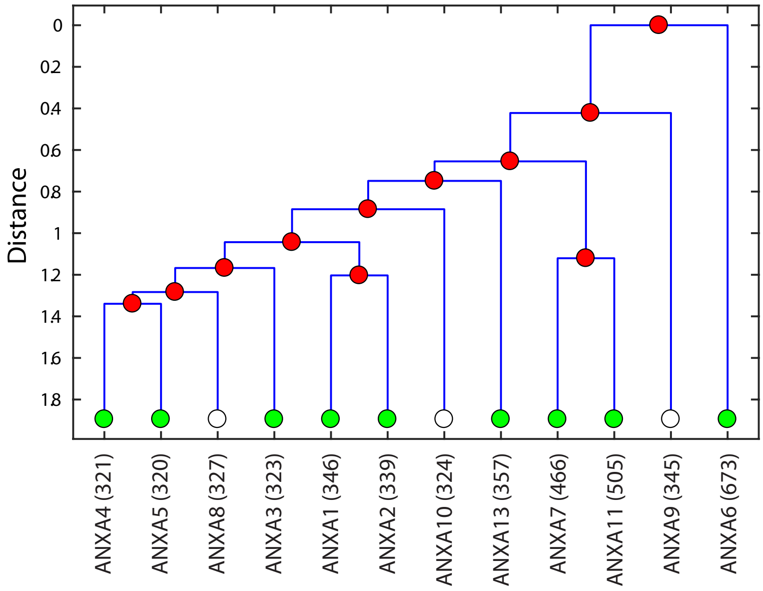

Figure S11: Dendrogram for annexins made with the WPGMA method for comparison with the dendrogram in Fig. 7e made with the UWPGMA method. The two dendrograms have identical connections. See code in Fig. S10 and Methods section for details.

References

[1] W. Helfrich, Elastic properties of lipid bilayers: theory and possible experiments, Z. Naturforsch 28 (1973) 693–703. doi:10.1515/znc-1973-11-1209.

[2] D. Marsh, Handbook of lipid bilayers, CRC press, 2013.

[3] W. Pezeshkian, A. G. Hansen, L. Johannes, H. Khandelia, J. Shillcock, P. S. Kumar, J. Ipsen, Membrane invagination induced by Shiga toxin B-subunit: from molecular structure to tube formation, Soft matter 12 (23) (2016) 5164–5171. doi:10.1039/c6sm00464d.

[4] W. Pezeshkian, L. J. Naabo, J. H. Ipsen, Cholera toxin B subunit induces local curvature on lipid bilayers, FEBS open bio 7 (11) (2017) 1638–1645. doi:10.1002/2211-5463.12321.

[5] T. L. Boye, K. Maeda, W. Pezeshkian, S. L. Sonder, S. C. Haeger, V. Gerke, A. C. Simonsen, J. Nylandsted, Annexin A4 and A6 induce membrane curvature and constriction during cell membrane repair, Nature Comm. 8 (1623). doi:10.1038/s41467-017-01743-6.

[6] E. Evans, D. Needham, Attraction between lipid bilayer membranes in concentrated solutions of nonadsorbing polymers: comparison of mean-field theory with measurements of adhesion energy, Macromolecules 21 (6) (1988) 1822–1831. doi:10.1021/ma00184a049.

[7] T. Yeung, G. E. Gilbert, J. Shi, J. Silvius, A. Kapus, S. Grinstein, Membrane phosphatidylserine regulates surface charge and protein localization., Science (New York, N.Y.) 319 (5860) (2008) 210–3. doi:10.1126/science.1152066.
